# Robustification of a One-Dimensional Generic Sigmoidal Chaotic Map with Application of True Random Bit Generation

**DOI:** 10.3390/e20020136

**Published:** 2018-02-20

**Authors:** Nattagit Jiteurtragool, Tachibana Masayoshi, Wimol San-Um

**Affiliations:** 1 School of Systems Engineering, Electronic and Photonic Engineering, Kochi University of Technology, Tosayamada, Kami City, Kochi 782-8502, Japan; 2 Center of Excellence in Intelligent Systems Integration, Faculty of Engineering, Thai-Nichi Institute of Technology (TNI), 1771/1, Pattanakarn Rd, Suan Luang, Bangkok 10250, Thailand

**Keywords:** robustification, unification, linearization, chaotic map, sigmoid, robust chaos, true random bit generator

## Abstract

The search for generation approaches to robust chaos has received considerable attention due to potential applications in cryptography or secure communications. This paper is of interest regarding a 1-D sigmoidal chaotic map, which has never been distinctly investigated. This paper introduces a generic form of the sigmoidal chaotic map with three terms, i.e., *x_n_*_+1_ = ∓*Af*_NL_(*B*x*_n_*) ± *C*x*_n_* ± *D*, where *A*, *B*, *C*, and *D* are real constants. The unification of modified sigmoid and hyperbolic tangent (tanh) functions reveals the existence of a “unified sigmoidal chaotic map” generically fulfilling the three terms, with robust chaos partially appearing in some parameter ranges. A simplified generic form, i.e., *x_n_*_+1_ = ∓*f*_NL_(*B*x*_n_*) ± *C*x*_n_*, through various S-shaped functions, has recently led to the possibility of linearization using (i) hardtanh and (ii) signum functions. This study finds a linearized sigmoidal chaotic map that potentially offers robust chaos over an entire range of parameters. Chaos dynamics are described in terms of chaotic waveforms, histogram, cobweb plots, fixed point, Jacobian, and a bifurcation structure diagram based on Lyapunov exponents. As a practical example, a true random bit generator using the linearized sigmoidal chaotic map is demonstrated. The resulting output is evaluated using the NIST SP800-22 test suite and TestU01.

## 1. Introduction 

In 1993, Majumdar and Mitra [1] first coined the phrase “*robust chaos*” in dynamic optimization models represented by a quadratic map family. Later in 1996, a search for robust chaos in a discrete-time neural network was conducted by R. Dogaru et al. [2] to discover a compact set of parameters, included in a weight space, that could sustain chaotic behaviors but remain unchanged. In 1998, S. Banerjee et al. [3] defined robust chaos as “*the absence of periodic windows and coexisting attractors in some neighborhood of the parameter space.*” Such a definition implies that any changes or variations in system parameters would not result in the fragility of chaos. A practical example of robust chaos in a 2-D piecewise smooth system was also demonstrated through a current-mode controlled boost converter.

A search for robust chaos generation approaches has been of considerable interest due to the suitability of robust chaos in practical applications in science and engineering, such as cryptography and secure communications [4,5,6,7,8,9]. Andrecut and Ali [10,11] reconstructed 2-D smooth unimodal maps via non-integer powers for robust chaos by means of mapping a critical point into an unstable fixed point that was not in the basin of attraction of a periodic attractor where, consequently, no periodic attractors occurred. G. Perez [12] has further analyzed the linear interpolation between fully chaotic logistic and quartic maps suggested by S. Thomae, and the results reveal a bifurcation diagram without any periodic windows. 

Recently, several approaches to the generation of robust chaos have been reported, involving techniques relating to (i) the determination of critical behavior of the Lyapunov exponent near the transition to robust chaos via type-III intermittency for a 1-D singular map [13], (ii) two methods for a prescribed invariant measure and varying Lyapunov exponent as well as a prescribed constant invariant measure and varying Lyapunov exponent [14], (iii) a structural synthesis of a state space energy-based adaptive controller [15], (iv) the basis of symmetry violations in attractors [16], and (v) the invariant center manifold [17]. 

In 2012, the open problem on “*Is a unifying chaotic dynamic system possible?*” was raised by Z. Elhadj and J. C. Sprott [18], and a multifunction mathematical model, a so-called unified chaotic map, was proposed with the capability of generating hyperbolic, Lorenz-type, and quasi-attractors [19]. J.C. Sprott [20] also introduced a particular 2-D unified piecewise smooth map that contained Hénon and Lozi maps. It is remarkable to note that the unification of a piecewise smooth map could exhibit robust chaos in some portions of a bifurcation parameter region, which is, in fact, a transition between Hénon and Lozi maps.

In accordance with [19,20], it is natural to wonder whether there is a possibility of the unification of a category of simple 1-D smooth chaotic maps that can generate robust chaos. Exhaustive searches and investigations into a family of S-shaped functions have led to a generic form for a smooth sigmoidal chaotic map, presented in this paper. The unification and simplification of the generic smooth sigmoidal chaotic map will be discussed. The linearization of a simplified smooth sigmoidal chaotic map using either the hardtanh or the signum function potentially exhibits robust chaos over an entire range of parameters. Chaos dynamics will be described in terms of apparent time-domain chaotic waveforms and their histogram, cobweb plots, frequency spectrum, equilibria, Jacobian, bifurcation structure diagram based on Lyapunov exponents, bifurcation diagram, and recurrence plot (RP). As for practical examples, a true random bit generator (TRBG) with statistical tests results from the NIST SP800-22 test suite and TestU01 using the linearized sigmoidal chaotic map will be demonstrated.

## 2. Generic One-Dimensional Sigmoidal Chaotic Maps

### 2.1. Unification of Generic Sigmoidal Chaotic Map

The proposed unification process commences by considering a generic sigmoidal chaotic map, which can be preliminarily defined by the recurrence relation of the form

(1) $$x_{n+1}=\mp Af_{\mathrm{NL}}(Bx_{n})\pm Cx_{n}\pm D,$$

where *x_n_* is a real variable, *f*_NL_(*x_n_*) is a sigmoidal function, and the parameters *A*, *B*, *C*, and *D* are real constants. With reference to (1), this paper initially considers a typical sigmoid function, which exhibits S-shaped transfer function characteristics within the range (0, 1) throughout an entire domain (−∞, +∞). In other words, a mathematical model is *f*(*x*) = 1/(1 + exp(−*x*)). Nonetheless, the substitution of the sigmoid function as *f*_NL_(*x_n_*) in (1) could not induce chaos. Therefore, this paper realizes a modified sigmoid function *f*_mod_(*x*) as follows:

(2) $$f_{\mathrm{mod}}(x)=2\left(\frac{1}{1+e^{-x}}\right)-1$$

It is seen in (2) that the range of *f*_ms_(*x*) is a typical sigmoid function where the function is doubled and shifted down to be (−1, 1). Notice that the nonlinearity in (2) apparently associates to a hyperbolic tangent (tanh) function, i.e.,

(3) $$f(x)=\tan h\;(x)=\frac{1-e^{-2x}}{1+e^{-2x}}=2\left(\frac{1}{1+e^{-2x_{n}}}\right)-1$$

Note that the constant 2 is essential as a result of the mathematical transformation. Realizing functions (2) and (3) in the generic sigmoidal chaotic map results in a unified sigmoidal chaotic map that contains modified sigmoid and tanh functions given by

(4) $$x_{n+1}=\pm 2\left(\frac{1}{1+e^{-Bx_{n}}}\right)\mp Cx_{n}\mp 1$$

It is clear that Equation (4) provides three complete mathematical terms to the generic sigmoidal chaotic map described in (1), where parameters *A* and *D* are 2 and 1, respectively, while parameters *B* and *C* are assigned as bifurcation parameters. Equation (4) also comprises a conjugate of two unified sigmoidal chaotic maps as follows:

(5) $$x_{n+1}=2\left(\frac{1}{1+e^{-Bx_{n}}}\right)-Cx_{n}-1$$

(6) $$x_{n+1}=-2\left(\frac{1}{1+e^{-Bx_{n}}}\right)+Cx_{n}+1$$

**Figure 1:**
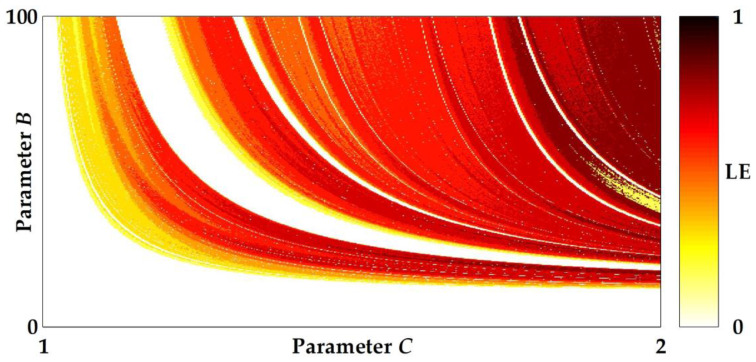
Plots of a bifurcation structure of parameters *C* versus *B* of the unified sigmoidal chaotic map in (5), where the heat diagram indicates a positive Lyapunov exponent. LE = Lyapunov exponent.

In order to investigate the chaotic dynamics of the unified sigmoidal chaotic maps, the Lyapunov exponent (LE) is calculated. The LE is defined as a quantitative measure that characterizes the rate of separation of infinitesimally close trajectories, and can be described as

(7) $$\mathrm{LE}=\underset{N\to \infty }{\lim}\frac{1}{N}\sum_{n=1}^{N}\log_{2}\frac{dx_{n+1}}{dx_{n}}$$

where N is the number of iterations. A positive LE typically indicates chaotic behaviors, and a larger value of LE results in a higher degree of chaoticity. The LEs of the system are calculated by using 100,000 iterations of data. Figure 1 illustrates plots of a bifurcation structure of parameters *C* versus *B* of the unified sigmoidal chaotic map in (5), where the heat diagram indicates a positive LE and a white color represents a non-chaotic region while the black color represents the maximum LE of 1. The shading means that the LE increases correspondingly from yellow to red. Within the region of parameters 0 < *B* < 100 and 1 < *C* < 2, the white color roughly indicates where LE ≤ 0, and it appears in a few regions. However, there is some partial portion of parameter space that appears to be robust.

**Figure 2:**
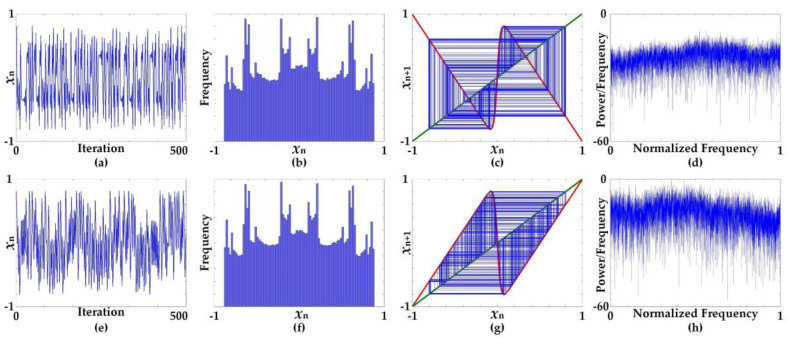
Characteristics of chaotic waveforms in time domain and plots of histogram, cobweb, and frequency spectrum using periodogram at specific parameters *B* = 75 and *C* = 1.9; (**a**–**d**) characteristics of Equation (5), (**e**–**h**) characteristics of Equation (6).

Figure 2 shows the characteristics of time-domain chaotic waveforms and the histogram, cobweb, and frequency spectrum using periodogram plots at specific parameters *B* = 75 and *C* = 1.9, arbitrarily selected from the chaotic region. The waveforms in the time domain are apparently chaotic but are slightly different. The histograms for both Equations (5) and (6), obtained from 100,000 iterations, are very similar. However, the characteristics of the cobweb plots are significantly different. Equation (5) exhibits a superimposed square pattern, while Equation (6) reveals a hexagon pattern. It can be seen from the frequency spectrum that both Equations (5) and (6) offer a flat spectrum feature.

### 2.2. Simplification of Generic Sigmoidal Chaotic Map

With reference to a generic sigmoidal chaotic map in (1), it is also possible to simplify a mathematical model through the utilization of other S-shaped nonlinear functions through the specific parameters *A* = 1 and *D* = 0. In other words, the simplified generic sigmoidal chaotic map is

(8) $$x_{n+1}=\mp f_{\mathrm{NL}}(Bx_{n})\pm Cx_{n}$$

Table 1 summarizes six simplified chaotic maps based on (8), the results of utilizing nonlinear functions *f*_NL_(*x*) with S-shaped transfer function characteristics. With respect to the mathematical aspects, the cases NM_1_, NM_2_, and NM_3_ are based on inverse trigonometric properties. Meanwhile, the case NM_4_ is a special function in the form of an integral, which is originally derived from a Gaussian function, while NM_5_ and NM_6_ are special differentiable algebraic functions.

**Figure 3:**
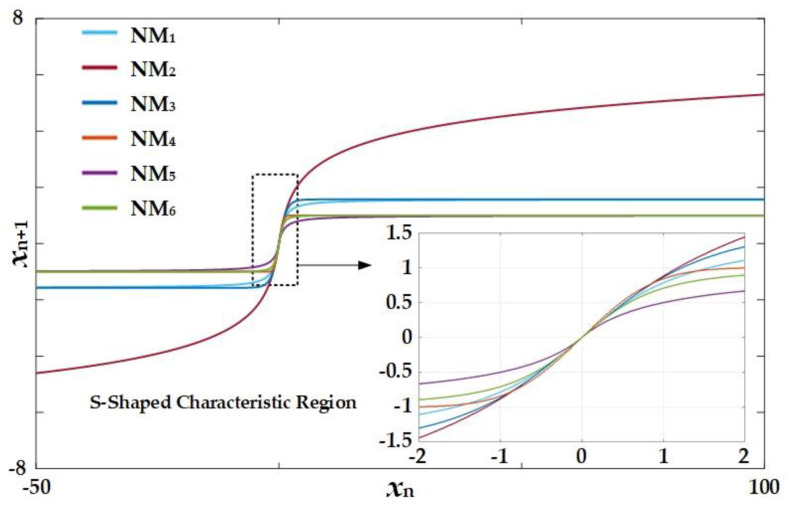
Plots of transfer function characteristics of the nonlinear functions of the cases NM_1_ to NM_6_.

In order to investigate and compare S-shaped transfer function characteristics, Figure 3 depicts plots of transfer functions of the six nonlinear functions. It is apparent that only NM_4_ has a range in the y-axis in the region (−1, 1), which closely resembles nonlinearity in a unified sigmoidal chaotic map, whereas the range of NM_2_ appears to be (−∞, +∞). The ranges of the four remaining cases are limited at certain specific levels. This phenomenon implies that the S-shaped nonlinearity that plays an important role in inducing chaos occurs in a short domain of approximately (−2, 2), and, therefore, the parameter *B*, which was introduced in the generic sigmoidal chaotic map, consequently becomes a significant factor in determining the chaos dynamics.

## 3. Linearization of Simplified Sigmoidal Chaotic Map for Robust Chaos

Regarding (8), rather than utilizing any S-shaped nonlinear functions, the linearized sigmoidal functions including the hardtanh and signum functions are employed. In other words, the proposed linearized sigmoidal chaotic maps are as follows:

(9) $$x_{n+1}=\mp \mathrm{hardtanh}\;(Bx_{n})\pm Cx_{n}$$

(10) $$x_{n+1}=\mp \mathrm{sgn}\;(Bx_{n})\pm Cx_{n}$$

where the hardtanh and signum are defined as

(11) $$\mathrm{hardtanh}\;\left(x\right)=\{\begin{array}{rr} -1; & x<-1\\ x; & -1\leq x\leq 1\\ 1; & x>1 \end{array}$$

(12) $$\mathrm{sgn}\;\left(x\right)=\{\begin{array}{cc} -1; & x<-1\\ 0; & x=0\\ 1; & x>1 \end{array}=\{\begin{array}{cc} \frac{x}{\left|x\right|}; & x\neq 0\\ 0; & x=0 \end{array}$$

The linearized sigmoidal chaotic map based on the hardtanh function in (9) is a conjugate of two chaotic maps, i.e.,

(13) $$x_{n+1}=\mathrm{hardtanh}\;(Bx_{n})-Cx_{n}$$

(14) $$x_{n+1}=-\mathrm{hardtanh}\;(Bx_{n})+Cx_{n}$$

Meanwhile, the linearized sigmoidal chaotic map for (10), based on the signum function, is also the conjugate of two chaotic maps and can be expressed as

(15) $$x_{n+1}=\mathrm{sgn}\;(Bx_{n})-Cx_{n}$$

(16) $$x_{n+1}=-\mathrm{sgn}\;(Bx_{n})+Cx_{n}$$

In order to investigate the chaotic dynamic of the linearized sigmoidal chaotic maps, the Jacobian of the linearized sigmoidal chaotic map, which can be calculated through a first derivative as |J(*x_n_*)| = *f*’(*x_n_*), is considered. Typically, the discrete time system becomes unstable in the condition of |J(*x_n_*)| > 1, while the chaotic map needs to operate under an unstable condition in order to induce the chaos. With reference to (9), the unstable region of the linearized sigmoidal chaotic maps based on the hardtanh function, which is the parameter region where the chaos can occur, is calculated and provides the following result,

(17) |C|>1∪|C−B|>1

whereas the unstable region of the linearized sigmoidal chaotic maps based on the signum function in (10) is calculated and results in

(18) |C|>1

**Figure 4:**
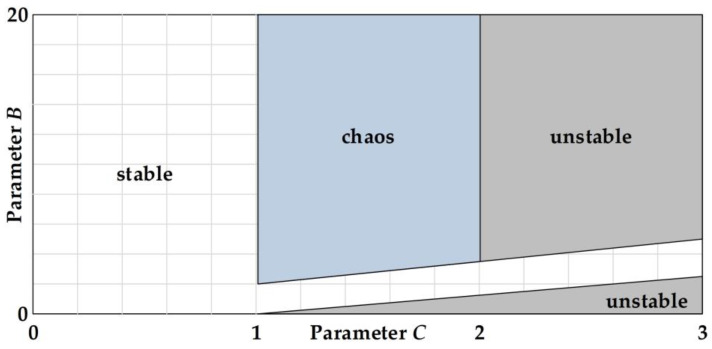
The plots of unstable and chaos regions with reference to (17), where the regions in grey and blue represent the unstable region and the chaos region, respectively.

**Figure 5:**
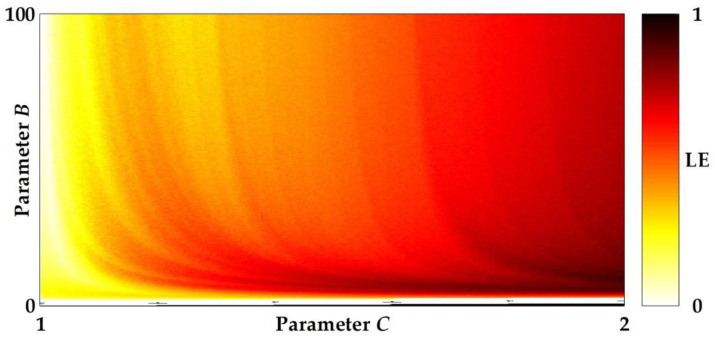
Plots of a bifurcation structure of parameter *C* versus *B* of the hardtanh-based linearized sigmoidal chaotic map in (13), where the heat diagram indicates a positive Lyapunov exponent.

**Figure 6:**
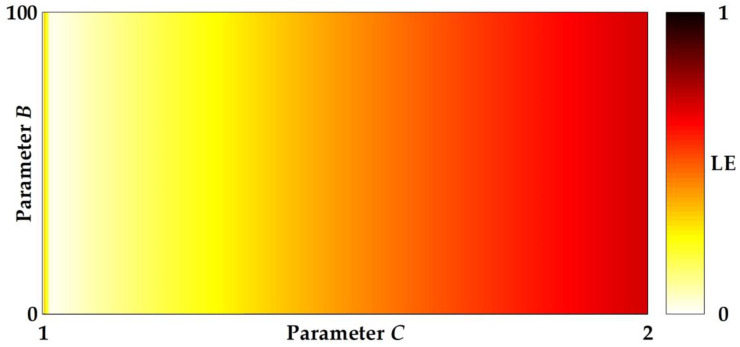
Plots of a bifurcation structure of parameters *C* versus *B* of the signum-based linearized sigmoidal chaotic map in (15), where the heat diagram indicates a positive Lyapunov exponent.

Within the region of parameters 0 < *B* < 15 and 1 < *C* < 3, Figure 4 depicts the plots of the unstable and chaos regions, where the grey region represents the unstable region regarding (17), while the chaos region, which is considered a subset of the unstable region, is represented by the blue region. The chaos region in Figure 4 corresponds to the plots of the bifurcation structure of parameters *C* versus *B* with regard to the linearized sigmoidal chaotic maps based on the hardtanh function in (13), as shown in Figure 5. Nonetheless, the plots of the bifurcation structure of parameters *C* versus *B* for the linearized sigmoidal chaotic map based on the signum function in (15), which is illustrated in Figure 6, shows the exact same values of LE for any values of *B* with respect to the signum function in (12). It is noticeable that the bifurcation structure in Figure 5 and Figure 6 illustrate the results according to the unstable regions in (17) and (18), respectively.

**Figure 7:**
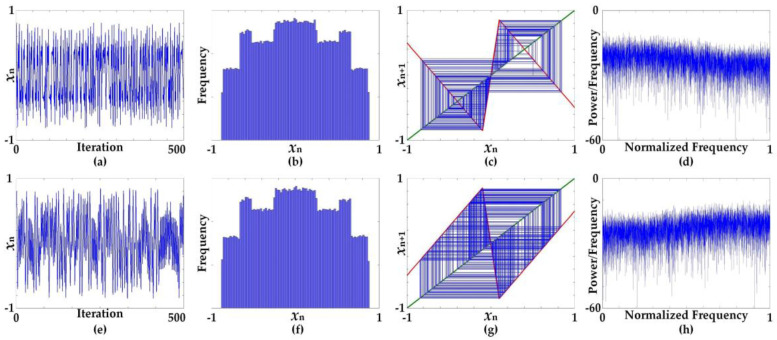
Characteristics of chaotic waveforms in time domain and plots of histogram, cobweb, and frequency spectrum using periodogram at specific parameters *B* = 15 and *C* = 1.9; (**a**–**d**) characteristics of Equation (13), (**e**–**h**) characteristics of Equation (14).

**Figure 8:**
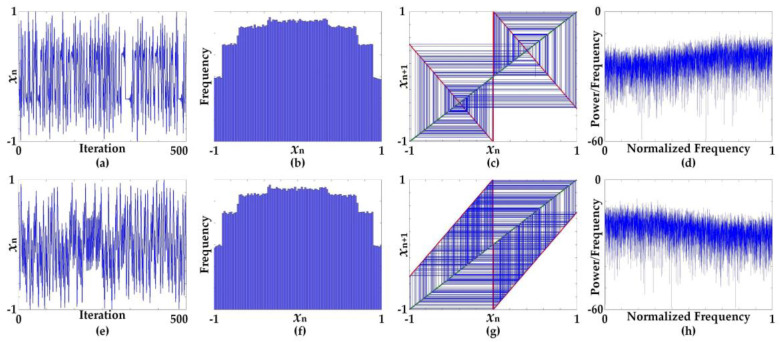
Characteristics of chaotic waveforms in time domain and plots of histogram, cobweb, and frequency spectrum using periodogram at specific parameter *B* = 1 and *C* = 1.9; (**a**–**d**) characteristics of Equation (15), (**e**–**h**) characteristics of Equation (16).

The chaotic map, considered the system *x_n_*_+1_ = *f*(*x_n_*), typically has a point where x* = *f*(x*) and is considered a fixed point (equilibrium). Table 2 summarizes the fixed points of the linearized sigmoidal chaotic maps based on the hardtanh function in (13) and (14) and the signum function in (15) and (16), all of which appear to have three fixed points. Figure 7 and Figure 8 show the characteristics of the chaotic waveforms in the time domain as well as the histogram and cobweb plots at specific parameters, which were arbitrarily selected with regard to the chaotic regime, as seen in the bifurcation structure in Figure 5 and Figure 6. The characteristics of the cobweb plots are associated with the fixed points of the chaotic maps, as shown in Table 2. In the case where the fixed point is 0, it is a globally asymptotically stable point, as in |J(0)| = 0. The stability of the fixed point appears in the cobweb plot, where the inward spiral corresponds to the attraction of the stable fixed point, while the outward spiral corresponds to the repelling of the unstable fixed point. The complex closed loops in the cobweb represent a high period of orbit, which indicates an infinite number of non-repeating values. The cobweb plots also relate to the boundary values of *x_n_*_+l_, which depend upon the nonlinear term of the chaotic map, and for both cases of the linearized sigmoidal chaotic map in (9) and (10), the values of *x_n_*_+l_ fall into the region (−1, 1).

The linearized sigmoidal chaotic maps based on the hardtanh function offer robust chaos over the entire range of parameters where *B* > 3 and 1 < *C* < 2, while the linearized sigmoidal chaotic map based on the signum function shows robust chaos over the entire range of parameters where 1 *< C* < 2.

**Figure 9:**
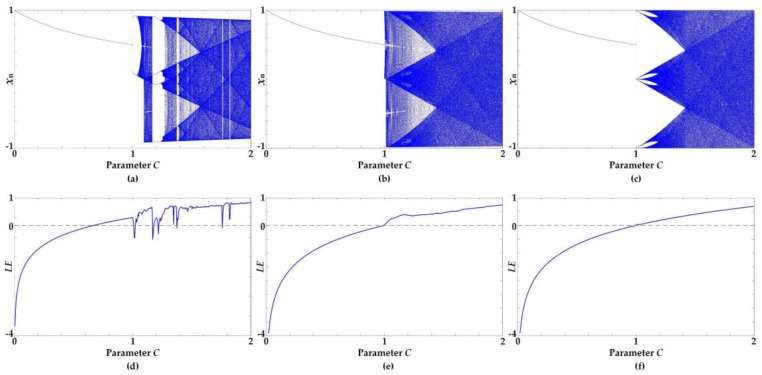
Plots of Bifurcation diagram and Lyapunov exponents (LEs) of chaotic maps at specific parameter *B* = 75; (**a**,**d**) the unified sigmoidal chaotic map in (5), (**b**,**e**) the hardtanh-based linearized sigmoidal chaotic map in (13), (**c**,**f**) signum-based linearized sigmoidal chaotic map in (15).

Other than the proposed measurement tool, the bifurcation diagram is employed as a tool for a qualitative measure. A plots bifurcation diagram and LEs were used to identify the chaotic behavior as well as the continuity of the proposed chaotic maps as shown in Figure 9. While parameter C is considered a bifurcation parameter, the bifurcation diagrams of the linearized sigmoidal chaotic maps in (13) and (15), as shown in Figure 9b–c, illustrate chaotic behavior over the entire range of parameters where 1 < C < 2, which corresponded to the LEs in Figure 9e–f. In other words, the linearized sigmoidal chaotic maps can offer robust chaos over the entire range of parameters. Conversely, the bifurcation diagrams and the LEs of the unified sigmoidal chaotic maps in (5) as shown in Figure 9a appear to have some periodic windows and illustrate intermittently chaotic behavior, which means the unified sigmoidal chaotic maps can only offer robust chaos for some partial portion of the parameter.

**Figure 10:**
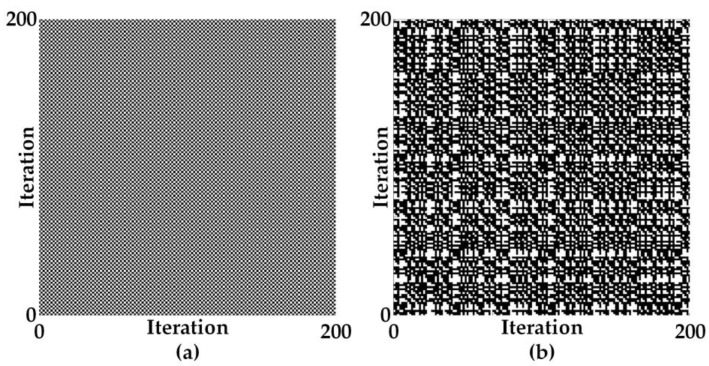
Recurrence plots of the signum-based linearized sigmoidal chaotic map in (15) for two different dynamic regimes, at specific parameter *B* = 1; (**a**) periodic regime: parameter *C* = 0.5, (**b**) chaotic regime: parameter *C* = 1.9.

The chaotic dynamics of the chaotic maps can also be described through a recurrence plot (RP) [21], as a typical random time series exhibits the RP with no structure while a periodic system causes the RP to exhibit some pattern. Figure 10 shows the RPs of the signum-based linearized sigmoidal for two different dynamic regimes. The purpose of the RP is to visualize the behavior of trajectories in phase space through a two-dimensional plot, which is especially beneficial in the case of high-dimensional systems. A dynamic system is represented by the trajectory (${\vec{x}}_{i}$) in *d*-dimensional phase space; hence, the recurrence plot, which can be viewed as the recurrence of a state at time *i* at a different time *j*, is defined by the matrix

(19) $$R_{i,j}=\Theta \left(\varepsilon -\left\|{\vec{x}}_{i}-{\vec{x}}_{j}\right\|\right),\;i,j=1,\ldots ,N,$$

where Θ(⋅) is the Heaviside function, *N* is the number of points ${\vec{x}}_{i}$, and ε is a threshold. Figure 9a illustrates the RP which appear to be a dot pattern as a result the system that operated in the periodic regime, while Figure 10b illustrates the RP while the system is operated in the chaotic regime which results in a RP with no structure.

## 4. True Random Bit Generation Based on the Proposed Linearized Sigmoidal Chaotic Map

### 4.1. Random Bit Generator

**Figure 11:**
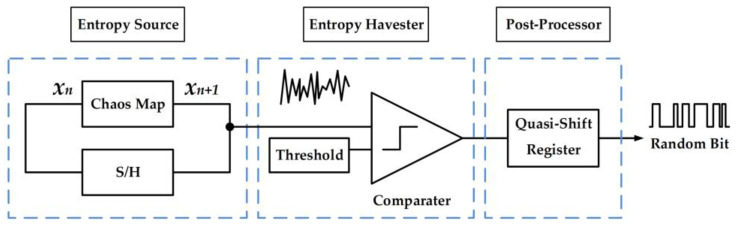
Proposed true random bit generator based on the signum-based linearized sigmoidal chaotic map.

The proposed true random bit generator (TRBG) is designed with respect to the typical structure of a true random bit generator, which consists of an entropy source, an entropy harvester, and a post-processor, as shown in Figure 11. The linearized sigmoidal chaotic map based on the signum function, which is driven by a sample and hold, is employed as the entropy source, and a comparator that acts as a 1-bit analog to the digital converter is considered the entropy harvester, while a quasi-shift register (QSR) is selected as the post-processor. 

#### 4.1.1. Entropy Source

Theoretically, a chaotic map is deterministic, which means that if the initial condition of a chaotic map is exactly known, the output behavior can be exactly predicted. However, chaotic maps in practical implementation operate without the initial condition by inherent noise of the system and are amplified in the positive gain feedback loop by the iteration of the output signal in the map function. The output of the chaotic map will be unpredictable and suitable for true random bit generation.

A chaotic map can typically be considered as an entropy source of true random bit generation [22,23,24] though the robustness of the chaotic map is a concern. The robust chaos means the absence of a periodic window, and the existence of the periodic windows in the range of parameters of the chaos region implies that a small variation of the parameters would remove the system from the chaotic regime and discontinue the chaotic behavior [25]. 

**Figure 12:**
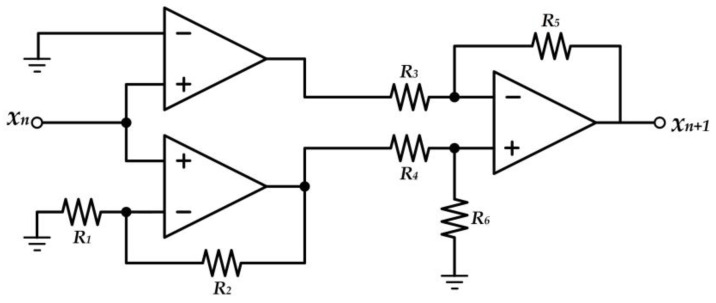
Circuit realizing the chaotic map with reference to the signum-based linearized sigmoidal chaotic map in (15).

Figure 12 shows the designed circuit of the chaotic map as the entropy source of the proposed TRBG, with reference to the signum-based linearized sigmoidal chaotic map in (15). The circuit consists of three operational amplifiers, (i) a comparator, (ii) a non-inverting operational amplifier, and (iii) a differential amplifier. The comparator operational amplifier is employed as the signum function, and it can be defined as

(20) $$\mathrm{comp}(V+)=\{\begin{array}{cc} +Vcc; & V+>V-\\ -Vcc; & V+<V- \end{array}$$

where V+ and V− are the inverting and non-inverting input of the comparator, respectively. The V+ can be considered an input *x*_n_ of the signum function, as a result of specifying the V−, which is a reference voltage of the comparator, as 0. In order for the comparator to perform as the signum function, the circuit is supplied with +1V and −1V as +V*cc* and –V*cc*, respectively.

Regarding the chaotic map in (15), the input *x_n_* is amplified by the non-inverting operational amplifier gain, as V_out_ = V_in_(1 + R_2_/R_1_), where R_3_, R_4_, R_5_, and R_6_ are set to be equal. The subtraction of the output of the comparator from the amplified input *ax_n_* results in the output of the chaotic map, *x_n_*_+1_.

#### 4.1.2. Entropy Harvester

The comparator with threshold *T*, which is the entropy harvester, as shown Figure 11, digitizes the generated signal from the chaotic map; this can be expressed mathematically as

(21) $$\mathrm{com}(x)=\{\begin{array}{cc} 0; & x\leq T\\ 1; & T\leq x \end{array}$$

The threshold is carefully chosen in order to generate numbers with a high level of randomness, or in other words, to harvest the entropy where it is at its maximum. Shannon’s entropy is defined as

(22) $$H=-\sum_{i=0}^{1}P_{i}\log_{2}P_{i}$$

**Figure 13:**
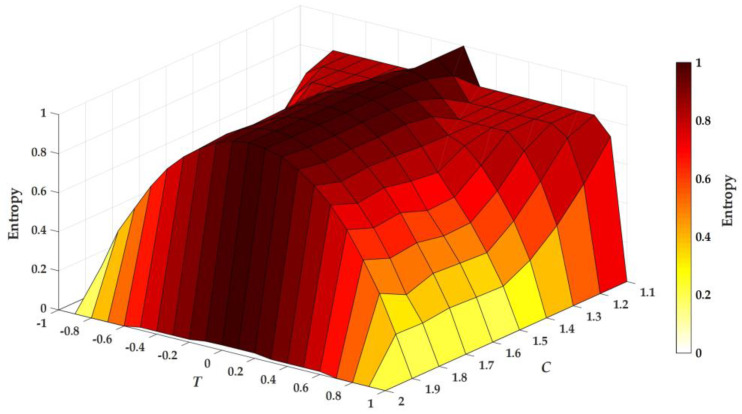
Plots of entropy versus threshold *T* and parameter *C* of the signum-based linearized sigmoidal chaotic map in (15).

The entropy is calculated over the entire range of parameters, where 1 < *C* < 2, resulting in the three-dimensional plot in Figure 13. The entropy is plotted versus the threshold value and parameter *C* of the signum-based linearized sigmoidal chaotic map in (15); note that the maximum entropy can be achieved when the threshold value is at 0 for any value parameter *C* in the chaotic regime. 

#### 4.1.3. Post-Processor

**Figure 14:**
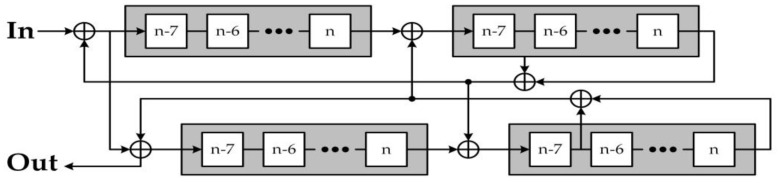
Structure of the quasi-shift register-based post-processor.

Even though the result from the entropy harvester is a random bit sequence, the post-processor is still required to improve the statistical imperfections of the generated sequences. Although there are many post-processing methods, the quasi-shift register was selected as the post-processor in the proposed TRBG due to its simple structure [26], with only a single input required, and its property of reducing the imperfection of the random bit sequences while still maintaining its generation rate. The structure of the quasi-shift register comprises four shift registers, A, B, C, and D, with a selected length *n* = 8, as depicted in Figure 14. The post-processor initially starts by memorizing the generated bit from the TRBG into the shift register A, and then it performs XOR operation between shift registers. These processes are repeated several times in order to increase the complexity of the bit sequence.

### 4.2. Randomness Performance Evaluation

#### 4.2.1. NIST SP800-22 Test Suite

The random bit sequence from the TRBG was examined according to the properties of a random sequence that can be described in terms of probability. Although there are a variety of statistical tests, the NIST SP800-22 test suite [27] is the statistical test most widely used to investigate the randomness of the output sequence from the TRBG. The NIST test suite, issued by the National Institute of Standards and Technology, is a statistical test package consisting of 15 tests; it is generally accepted as a standard test suite for any random number generators. The test can be used to examine the bit sequence by detecting a pattern of values that indicates the non-randomness (periodic) of the sequences, resulting in the probability values (*P*-values). The *P*-values for each test indicate a randomness of the bit sequences, and, typically, the test is considered to be passed for *P*-values greater than 0.01; otherwise, they are rejected.

The performance of the proposed TRBG was evaluated through the NIST statistical test suite with 100 Mbit data. The generated bit sequence is divided into 100 sequences with the length of 1Mbit for each block. The calculated *P*-values, as shown in Table 3, indicate that the proposed TRBG can pass all the tests.

#### 4.2.2. TestU01

TestU01 is a software library for statistically testing random bit generators [28]. The TestU01 library provides several test batteries, while each test battery also contains a collection of empirical statistical tests. Each statistical test can generate a *P*-value as well as the NIST test suite, which is considered as an indicator of passing the test. The test is considered passed if the generated *P*-value from the test falls into the interval [0.001, 0.999].

Three binary sequences with lengths 2^20^, 2^25^, and 2^30^ bits were generated from the proposed TRBG. The bit sequences were applied to the batteries Rabbit, Alphabit, and BlockAlphabit to evaluate the randomness. Each battery contains a different number of test. The Alphabit contains 17 statistical tests while the BlockAlphabit applies the Alphabit repeatedly to the reordered bits with the 6 different blocks sizes which are 1, 2, 4, 8, 16, and 32. In other words, the BlockAlphabit contains a total number of 17 × 6 = 102 statistical tests. The Rabbit applies 38, 39, and 40 test to the bit sequence with lengths 2^20^, 2^25^, and 2^30^ bits, respectively. The results of the TestU01 are presented in Table 4. The proposed TRBG can pass all the tests.

## 5. Conclusions

In this paper, the unified and simplified forms of the generic sigmoidal chaotic map and the linearized sigmoidal chaotic map were presented. Chaos dynamics were described in terms of chaotic waveforms, histogram, cobweb plots, fixed point, Jacobian, and a bifurcation structure diagram based on Lyapunov exponents; these revealed that both hardtanh function-based and signum function-based linearized sigmoidal chaotic maps have the potential to offer robust chaos over the entire range of parameters. In other words, it can be summarized that based on a linearized sigmoidal, the proposed sigmoidal chaotic map can offer robust chaos over the entire range of parameters. The true random bit generator based on the linearized sigmoidal chaotic map was demonstrated as a practical example; hence, the robust chaotic map is suitable as an entropy source. The resulting random bit sequence passed the NIST statistical test suite and the TestU01. Performance test results from both statistical tests show that the proposed linearized sigmoidal chaotic maps are suitable for application such as a TRBG.

## Figures and Tables

**Table 1:** Summary of six simplified sigmoidal chaotic maps involving nonlinear functions *f*_NL_(*x*) with S-shaped transfer function characteristics.

Cases	Descriptions	*f*_NL_(*x*) with No Parameters	Chaotic Maps
NM_1_	Inverse Tangent Function	$f_{\mathrm{NL}1}(x)=\tan{}^{-1}\left(x\right)$	$x_{n+1}=\mp \tan{}^{-1}(Bx_{n})\pm Cx_{n}$
NM_2_	Inverse Hyperbolic Sine Function	$f_{\mathrm{NL}2}(x)=\sin h{}^{-1}\left(x\right)$	$x_{n+1}=\mp \sin h{}^{-1}(Bx_{n})\pm Cx_{n}$
NM_3_	Gudermannian Function	$f_{\mathrm{NL}3}(x)=\tan{}^{-1}\left(\sinh\;\left(x\right)\right)$	$x_{n+1}=\mp \tan{}^{-1}(\sin h\;(Bx_{n}))\pm Cx_{n}$
NM_4_	Error Function	$f_{\mathrm{NL}4}(x)=\frac{2}{\sqrt{\pi }}\int_{0}^{x}e^{-t^{2}}dt$	$x_{n+1}=\mp \frac{2}{\sqrt{\pi }}\int_{0}^{Bx}e^{-t^{2}}dt\pm Cx_{n}$
NM_5_	Soft Signum Function	$f_{\mathrm{NL}5}(x)=\frac{x}{1+\left|x\right|}$	$x_{n+1}=\mp \frac{Bx_{n}}{1+\left|Bx_{n}\right|}\pm Cx_{n}$
NM_6_	Specific Algebraic Function	$f_{\mathrm{NL}6}(x)=\frac{x}{\sqrt{1+x^{2}}}$	$x_{n+1}=\mp \frac{Bx_{n}}{\sqrt{1+{\left(Bx_{n}\right)}^{2}}}\pm Cx_{n}$

**Table 2:** Summary of the fixed points of the linearized sigmoidal chaotic maps.

Chaotic Map Equations	x* = *f*(x*)	Fixed Points x*
(10)	x*=hardtanh (Bx*)−Cx*	$0,\;\frac{1}{C-1}$ and $-\frac{1}{C-1}$
(11)	x*=−hardtanh (Bx*)+Cx*	$0,\;\frac{1}{C+1}$ and $-\frac{1}{C+1}$
(12)	x*=sgn (Bx*)−Cx*	$0,\;\frac{1}{C-1}$ and $-\frac{1}{C-1}$
(13)	x*=−sgn (Bx*)+Cx*	$0,\;\frac{1}{C+1}$ and $-\frac{1}{C+1}$

**Table 3:** National Institute of Standards and Technology (NIST) statistical test suite.

Test Methods	*P*-Value	Proportion	Result
Frequency (monobit)	0.7981	0.99	Pass
Block Frequency	0.5544	0.99	Pass
Runs	0.6163	1.00	Pass
Longest Run	0.7399	1.00	Pass
Binary Matrix Rank	0.2133	1.00	Pass
Discrete Fourier Transform	0.7791	1.00	Pass
Non-overlapping Template Matching	0.4980	0.99	Pass
Overlapping Template Matching	0.9114	0.98	Pass
Universal Statistical	0.7597	0.99	Pass
Linear Complexity	0.6579	0.99	Pass
Serial	0.4983	0.98	Pass
Approximate Entropy	0.3669	1.00	Pass
Cumulative Sums	0.5139	0.99	Pass
Random Excursions	0.3322	0.98	Pass
Random Excursions Variant	0.3384	0.99	Pass

**Table 4:** TestU01. TRBG = true random bit generator.

Random Bit Generator	Test Batteries		
	Rabbit	Alphabit	BlockAlphabit
2^20^ bits			
Proposed TRBG	38/38	17/17	102/102
2^25^ bits			
Proposed TRBG	39/39	17/17	102/102
2^30^ bits			
Proposed TRBG	40/40	17/17	102/102
